# Strategies for Microbial Decontamination of Fresh Blueberries and Derived Products

**DOI:** 10.3390/foods9111558

**Published:** 2020-10-28

**Authors:** Liliana Pérez-Lavalle, Elena Carrasco, Antonio Valero

**Affiliations:** 1 Faculty of Basic and Biomedical Sciences, Universidad Simón Bolívar, Barranquilla 080002, Colombia; 2 Department of Food Science and Technology, International Campus of Excellence in the AgriFood Sector (CeiA3), University of Córdoba, 14014 Córdoba, Spain; elena.carrasco@uco.es (E.C.); avalero@uco.es (A.V.)

**Keywords:** blueberry, processed fruits, non-thermal technologies, chemical strategies, physical strategies, biological strategies, microbial decontamination, quality and safety

## Abstract

Increasing consumption of blueberries is associated with appreciation of their organoleptic properties together with their multiple health benefits. The increasing number of outbreaks caused by pathogenic microorganisms associated with their consumption in the fresh state and the rapid spoilage of this product which is mainly caused by moulds, has led to the development and evaluation of alternatives that help mitigate this problem. This article presents different strategies ranging from chemical, physical and biological technologies to combined methods applied for microbial decontamination of fresh blueberries and derived products. Sanitizers such as peracetic acid (PAA), ozone (O_3_), and electrolyzed water (EOW), and physical technologies such as pulsed light (PL) and cold plasma (CP) are potential alternatives to the use of traditional chlorine. Likewise, high hydrostatic pressure (HHP) or pulsed electrical fields (PEF) successfully achieve microbial reductions in derivative products. A combination of methods at moderate intensities or levels is a promising strategy to increase microbial decontamination with a minimal impact on product quality.

## 1. Introduction

Blueberries (*Vaccinium* spp.) are fruits that are highly appreciated for their nutritional value and high concentration of bioactive substances such as vitamins, anthocyanins, and other phenolic compounds [1,2]. Several investigations have shown that the consumption of blueberries offers health benefits, such as their antioxidant and anticancer effect and protection against cardiovascular diseases and diabetes [3,4,5,6]. However, despite their nutritional characteristics, the consumption of blueberries has been linked to foodborne outbreaks worldwide. In Connecticut in 1984, an outbreak of listeriosis was associated with the consumption of blueberries [7]; Calder et al. [8] reported an outbreak of hepatitis A in New Zealand in 2002 where raw blueberries were implicated; in 2006, blueberries and strawberries were identified as potential foods contaminated by non-O157 Shiga toxin-producing *Escherichia coli*, causing an outbreak in Massachusetts [9]; 14 cases of *Salmonella* Muenchen infection were linked to the consumption of fresh blueberries in 2009 [10]; in 2010, an outbreak of six cases of *Salmonella* Newport in Minnesota was attributed to the consumption of fresh blueberries [11]. Likewise, the presence of enteric pathogens in this product continues to be widely reported, such as norovirus and *Salmonella* in frozen blueberries [12,13].

Blueberries can be contaminated at any point in the production chain. The use of contaminated water and microbial contamination from pickers, food handlers, or equipment during harvest and post-harvest have been reported as prominent factors in the contamination of berries [14]. In this respect, some authors have studied the microbiological quality of blueberries and food contact surfaces, i.e., packing lines of fresh blueberries, and found that, out of 310 samples, 46 were positive for enterococci and 27 for faecal coliforms, pointing to these surfaces as potential sources of food contamination [15]. Furthermore, Quansah et al. [16] investigated the microbiological quality of fresh blueberries collected from six different packing houses, resulting in 1 positive sample for *Enterococcus* and 11 positive samples for faecal coliforms in three of these premises.

On the other hand, blueberries are very susceptible to deterioration due to water loss and mould growth. Blueberry rot is mainly caused by *Colletotrichum* spp., *Botrytis cinerea*, *Alternaria* spp., *Fusarium* spp., and *Penicillium* spp. [17,18,19]. In order to minimize the deterioration of blueberries, consideration should be given to the factors affecting the initial quality of the produce as well as the subsequent handling practices. These factors include the type of cultivar, cultural practices, growing environment and harvesting practices. Following harvesting, storage temperature, humidity and atmosphere are key in preventing early decay, and thus, in extending their shelf-life [20].

Most blueberries are destined for the fresh market; for example, in the United States, the world’s leading producer of blueberries, the per capita consumption of fresh blueberries in 2018 was higher than the consumption of frozen blueberries—0.49 kg and 0.09 kg, respectively [21]. Likewise, in Europe the demand for fresh blueberries has increased in recent years [22]. However, fresh blueberries are not subjected to any treatment for the eradication of pathogens, and no microbiological standards have been established for these products, which is a serious concern for food safety [16]. Blueberries for the fresh market are generally hand-picked, packaged, and kept at refrigeration temperatures to maintain their shelf-life [23,24]. Nevertheless, fresh blueberries destined to produce derived products such as juice, puree, and dehydrated products, as well as blueberries intended for freezing, are subjected to a sanitary treatment, which classically involves washing with chlorine. Despite the extended use of chlorinated water as a microbial decontamination treatment of fruits and vegetables, alternative microbial decontamination technologies and/or substances have emerged due to issues such as the limited antimicrobial efficacy of chlorine in the presence of organic matter, the formation of toxic compounds such as carcinogenic trihalomethane and chloramine, and its deleterious effect on organoleptic characteristics of fruits [25,26]. This has led to European countries such as Belgium, Denmark, Germany, and The Netherlands banning its use [27].

Based on consumer demand for safe food, several investigations have been conducted to develop and evaluate the effect of different antimicrobial strategies on fresh berries, with minimal impact on its quality and nutritional value (Appendix A Table A1). This review summarizes the studies published between 2010 and 2020 on chemical, physical, and biological strategies and the combination of these technologies applied to fresh blueberries and derived products for the reduction and inactivation of hazardous microorganisms and indigenous flora. Furthermore, effects on quality attributes of the products addressed by these studies are presented.

## 2. Methodology

A literature search was performed including published studies between 2010 and June 2020 in the Web of Science scientific database. The search words used were: (blueberr*) AND (*Salmonella* OR *Listeria monocytogenes* OR *Escherichia coli* O157:H7 OR murine norovirus OR norovirus OR hepatitis A OR Cyclospora OR total aerobic bacteria OR yeasts and moulds OR virus OR parasites) AND (chemicals OR solutions OR organic acids OR detergents OR inactivation OR antibacterial effect OR reduction OR decontamination OR sanitation OR disinfection). The search results were reviewed to select those corresponding to the application of microbial decontamination strategies in fresh blueberries and derived products. Additionally, the references of each article were reviewed to complement with other studies in the selected timespan.

## 3. Chemical Strategies

Chemical strategies are based on the use of different sanitizers or other compounds to contribute to the safety, quality, and shelf-life of foods of plant origin. As previously mentioned, novel microbial decontamination technologies have emerged mainly due to environmental and health problems and the limited antimicrobial efficacy of chlorine. Table A2 summarizes key aspects of published studies where different alternative chemical strategies to chlorine are applied for microbial decontamination of fresh blueberries. In following sections, relevant information concerning the different chemical strategies is presented.

### 3.1. Chlorine Dioxide

Chlorine dioxide (ClO_2_) has been studied as an alternative to chlorine due to its higher oxidizing capacity (2.5 times more than chlorine) [28]. In addition, its effectiveness is less pH dependent and produces fewer carcinogenic halogenated disinfection by-products (DBP) due to its low reaction with organic matter [29,30]. This compound inactivates microorganisms through the destabilization of cell membranes, interruption of protein synthesis, and oxidation of DNA/RNA/protein) [31]. ClO_2_ can be used both in its aqueous and gaseous form. However, several investigations have focused on the use of gaseous chlorine dioxide (gClO_2_) due to its high penetration, which allows it to reach inaccessible sites where microorganisms are attached [32]; likewise, the treated products do not require a subsequent washing as in the aqueous form, which is advisable for products susceptible to deterioration such as blueberries [33,34].

Among the limitations of ClO_2_ are the prolonged exposure times needed to obtain significant microbial reductions. Furthermore, the explosive nature of this compound limits its industrial application to some extent [31,35]. Other difficulties associated with the cost and on-site generation of gClO_2_ have been solved in recent years through the development of different methods that allow portable and low-cost generation [32].

Different studies have demonstrated the potential of gClO_2_ to reduce pathogens and native microbiota in fresh blueberries (Table A2). As an example, Zhang et al. [36] showed reductions of aerobic mesophilic bacteria and yeasts and moulds count (YMC) by >2 and >1 log CFU/g, respectively, after exposure to gClO_2_ (4 mg/L) for 12 h. In relation to the inactivation of bacterial pathogens, the cumulative gClO_2_ exposure of 1529 ppm-h achieved reductions of >3.8 log CFU/g of Shiga toxin-producing *E*. *coli* (STEC), *Listeria monocytogenes* and *Salmonella*. Likewise, the authors of that study demonstrated the advantage of using gClO_2_ under refrigeration temperatures (4 °C) [37]. Regarding inactivation of viruses, a ClO_2_ concentration of 0.63 ppm-h/g, achieved the reduction of Tulane virus (TV) in 3.82 log plaque forming units (PFU)/g [38]. It should be noted that most studies have focused on substitutes for human norovirus so there is a research gap in relation to other pathogens such as hepatitis A virus (HAV) which is also involved in foodborne outbreaks due to consumption of blueberries.

Some limitations of the use of gClO_2_ are related to the bleaching of fruits and vegetables when applied at high concentrations [31]. Kingsley et al. [39] reported that gClO_2_ produced from concentrations greater than 1 mg of NaClO_2_ may cause significant alterations in the appearance and quality of the samples. Chai et al. [37] reported that to avoid bleaching of the products, the concentration of ClO_2_ generated by the dry media method should not exceed 730 ppm (>1900 cumulative ppm-h in a 5-h treatment time). Additionally, processing factors such as exposure time, relative humidity and temperature can influence the antimicrobial efficacy of gClO_2_ [33]. Sun et al. [40] found a reduction in the efficacy of treatment after 6 days of storage at 10 °C, possibly due to the volatility of ClO_2_. In contrast, quality attributes such as firmness were maintained [40]. Kingsley et al. [39] also pointed out that the maintenance of relatively low and constant levels of ClO_2_ in combination with high relative humidity could improve the inactivation of the TV.

In relation to aqueous ClO_2_, the maximum concentration allowed in the United States for the treatment of fresh products is 3 ppm [34]. However, high concentrations are required to significantly reduce the microbial load of fruits and vegetables [33]. For blueberries, it is reported the reduction of initial populations of total aerobic bacteria and YMC in blueberries by 1.4–1.5 and 0.8–0.9 log CFU/g, respectively, by applying 100 ppm of ClO_2_ for 10 min. However, it should be noted that after treatment, blueberries stored at 4 °C for 12 days exhibited less deterioration and weight loss and better maintenance of the total anthocyanin content and sensory quality than samples stored at 20 °C storage [41]. Girard et al. [42] found that ClO_2_ (20 ppm for 1 min) was less effective in reducing norovirus murine in blueberries (<1 log reduction) compared to peroxyacetic acid (85 ppm) and sodium hypochlorite (50 ppm). To overcome the above drawbacks, combined methods are presented as a good alternative. In line with this, the combined treatment of aqueous ClO_2_ (2 ppm, 2 min) and UV (4 kJ/m^2^, 3 min) delayed the incidence of decay and improved the quality parameters, thus extending the post-harvest life of blueberries [43].

Overall, gClO_2_ appears as a promising alternative for the microbial decontamination of blueberries mainly due to its wide range of antimicrobial action, its high penetration power that allows access to irregular surfaces in blueberries, and the possibility of treatment under refrigerated conditions preventing breaks in the cold chain. The controlled release of this substance in sachets inside clamshells combined with refrigeration and high relative humidity also constitutes an alternative for maintaining the microbiological quality of blueberries destined for the fresh market. gClO_2_ dosages should be established to avoid bleaching effects and other quality defects on fruit. Therefore, it is recommended that microbial inactivation studies also evaluate the sensory parameters in blueberries during storage after treatment with gClO_2_.

### 3.2. Ozone

Ozone (O_3_) is a powerful oxidant GRAS (Generally Recognized As Safe) that spontaneously decomposes in oxygen. This compound exerts its antimicrobial action through the oxidation of cellular constituents such as proteins, lipids, and nucleic acids [35]. This can be applied to foods in their gaseous and aqueous forms [44]. However, ozone handling has some safety problems due to the risk of explosion and toxicity [45]. In addition, its excessive use can cause undesired organoleptic changes in the products and decompose phytochemical compounds. In this line, Bialka et al. [46] reported a slight darkening of blueberries after treatment with gaseous ozone.

Few studies on the antimicrobial action of ozone in blueberries have been carried out. With respect to the native microbiota, Concha-Meyer et al. [47] did not observe an inhibitory effect on moulds and yeasts in blueberries stored in an ozone atmosphere for 10 days with treatments of 4 ppm O_3_ at 4 °C or 2.5 ppm O_3_ at 12 °C. These treatments did not cause external damage to the fruits, while a reduction in weight loss and maintenance of firmness was evidenced during storage at 12 °C. In fact, similar to ClO_2_, O_3_ can inhibit the enzymatic activity which is responsible for maintaining firmness and reducing weight loss in some cultivars [40,47]. On the other hand, Jaramillo-Sanchez et al. [48] proposed a treatment with aqueous ozone at 18 ppm for 10 min to decrease fungal decomposition without affecting the weight loss of the fruits during refrigerated storage.

With regard to the effectiveness of ozone against pathogenic microorganisms on blueberries, Pangloli et al. [49] required an ozonated water treatment (1.5 mg/L, 5 min) to achieve a reduction of *E*. *coli* O157:H7 of 3.5 log CFU/g. However, other disinfectants tested, such as bleach solution (100 mg/L free chlorine), electrolyzed oxidizing water (30 mg/L free chlorine), and FIT^®^ solution, showed greater reductions. Concha-Meyer et al. [50] showed a 3 log reduction of *L*. *monocytogenes* in blueberries after 10 days of storage in an atmosphere of O_3_ (4 ppm) at 4 °C. On the other hand, compared to other disinfectants, Bridges et al. [51] showed higher reductions of closed circulated gClO_2_ than gaseous ozone for pathogens in blueberries (Table A2). The above findings indicate that high concentrations and exposure times of O_3_ are required to achieve significant microbial reductions.

The combination of O_3_ with other disinfection technologies could be further studied as an alternative to increase the antimicrobial capacity of this substance. For instance, a synergistic effect was observed by Kim et al. [52], who combined ozone vapor (4000 mg/L; 1 min) and ultraviolet light of 7.95 mW/cm^2^ intensity for 2 min, achieving a greater reduction of *E*. *coli* O157:H7 on blueberry calyx (3.05 log CFU/g) than the independent treatments.

It should be noted that none of the previous studies evaluated sensory parameters or content of bioactive compounds in blueberries immediately after treatment or during storage. Further research should be geared toward finding dosages that maximize microbial reductions without compromising the sensory and nutritional quality of blueberries.

### 3.3. Peracetic Acid

Peracetic acid (PAA) is another oxidizing agent, which results from the combination of peracetic acid and hydrogen peroxide. It is well-known as an environmentally friendly product as well as tolerant to pH, temperature and organic load [53]. PAA exerts its antimicrobial action through the production of reactive oxygen species that damage DNA and lipids. It can also denature proteins and affect the cell wall [54]. However, it has been reported that concentrations higher than those allowed (80 ppm) are necessary to achieve significant microbial reductions in fruits and vegetables—a distinct disadvantage [33].

Table A2 presents investigations that mainly demonstrate the efficacy of PAA against pathogens artificially inoculated in blueberries. For instance, Singh et al. [55] found that the application of PAA at 45 mg/L for 5 min achieved a reduction of 5.7 log CFU/g of *E*. *coli* O157:H7 in blueberries. Along these lines, the effect of PAA was comparable with other disinfectants such as chlorine-based sanitizers (acidic electrolyzed water, near-neutral electrolyzed water, and bleach) and lactic acid. In the case of *Salmonella*, the greatest reduction (6.4 log CFU/g) was achieved by the application of 2% lactic acid, followed by PAA at 45 and 100 mg/L, which resulted in the same reduction (5.9 log CFU/g). Experimental results on wash water demonstrated the elimination of pathogens by the application of both lactic acid and various PAA treatments, a very important finding for avoiding cross-contamination of products during the disinfection process [55].

In another study, Sheng et al. [56] demonstrated that the application of 0.4% Neo-Pure (PAA at 450 ppm) together with cold storage was more efficient in reducing *L*. *monocytogenes* compared to chlorine (100 ppm) (Table A2). In the study of Callahan et al. [57], in a custom-built pilot-scale processing line and setup for treatment of inoculated wild blueberries, *L*. *innocua* was reduced by 2.2 log CFU/g after immersion and spraying with PAA (80 ppm, 3 min). Furthermore, the importance of the inoculation method is highlighted, revealing that *L. monocytogenes* dip-inoculated on blueberries was more difficult to eradicate by both disinfectants than the spot-inoculated product [56].

Regarding the antimicrobial effect on the native flora, Sheng et al. [56] found that the use of Neo-Pure (2 min) followed by cold storage (4 °C and −15 °C) allowed the total plate count (TPC) to remain stable, with the exception of YMC at 4 °C, which increased up to ∼4.6 log CFU/g after 14 days of storage. On the other hand, a treatment of 3 min combining immersion in PAA (80 ppm) followed by spraying with chlorine (200 ppm) had limited success in reducing the yeast population (<1.0 log CFU/g) [57].

PAA is presented as a good alternative to chlorine for microbial decontamination of blueberries destined for the fresh and frozen market. However, more studies are required to design novel formulations based on combined strategies since the effect of PAA seems to be reduced against moulds according to recent studies. It would also be important to learn more about the subsequent microbial behaviour as well as the sensory and physicochemical parameters of blueberries during storage.

### 3.4. Hydrogen Peroxide and Organic Acids

Hydrogen peroxide (H_2_O_2_) and organic acids are presented as alternative means for protecting blueberries from deterioration and ensuring their safety. H_2_O_2_ is a strong oxidant that forms cytotoxic species that damage the proteins and DNA of microorganisms. It can be used both in aqueous and gaseous form at concentrations between 1–5% [45]; however, its use in fruits can be limited since the use of high concentrations could produce the oxidation of anthocyanins, changes in colour, and a decrease in antioxidant properties [33,58].

In contrast, the few studies reported on the use of H_2_O_2_ have shown its efficacy as a blueberry sanitizer without adversely impacting their quality. For example, blueberries treated with vapor phase H_2_O_2_ (60 min, up to 214 ppm of H_2_O_2_) exhibited an inactivation of murine norovirus (MNV) of 4 log PFU/mL. The treatment did not cause changes in the colour or consistency of the fruits [59]. Despite these studies, it would be advisable to examine more closely the organoleptic and nutritional aspects to obtain a better understanding of the actual efficiency of H_2_O_2_ for disinfection of blueberries.

On the other hand, the antimicrobial action of organic acids is due to the reduction of the environmental and cellular pH of microorganisms [60]. The main disadvantage of these treatments is the requirement of long exposure times (>5 min) to reduce the microbial load, which may affect the organoleptic characteristics of the products [29].

The use of single and combined washing methods of H_2_O_2_, organic acids (lactic acid, acetic acid and citric acid), and SDS has been applied for the reduction of *Salmonella* in blueberries [61]. The best treatments that achieved reductions ≥4.0 log CFU/g with an exposure time of 5 min were 0.5 mg/mL acetic acid plus 5000 ppm SDS, and 200 ppm H_2_O_2_ plus 5000 ppm SDS. These treatments showed equivalent reductions to 200 ppm of chlorine, and apart from the texture, they did not affect the colour, total phenolic (78.34 and 80.68 mg/100 g) and anthocyanin (0.324 and 0.308 mg/g) concentrations. In addition, the selected treatments kept the pathogen populations constant in blueberries and decreased the YMC during storage for 3 days at 4 °C.

On the other hand, due to the high redox potential, low pH and high concentration of dissolved active oxygen, electro-activated solutions of organic acid salts have been evaluated for microbial decontamination of blueberries [62]. These solutions, unlike electrolyzed NaCl solutions, have the advantage of not generating toxic chlorine. In this sense, electro-activated potassium acetate solution achieved significant reductions of moulds and pathogenic bacteria (Table A2). Likewise, in the same study [62], it was observed that the application of the solutions—potassium acetate, potassium citrate and calcium lactate—for 1 min, and subsequent storage at 2 °C for six weeks did not adversely affect the colour or texture of blueberries. Taking into account the advantages offered by electro-activated solutions of organic acid salts, it would be interesting to evaluate their potential in the inactivation of other pathogens such as viruses and parasites. Additionally, further evaluations on the effect of these on the bioactive compounds and organoleptic characteristics of blueberries at different concentrations and exposure times, would be of high interest.

### 3.5. Edible Coatings

The application of edible coatings has shown great potential in preserving the quality of fresh blueberries [63,64]. Edible coatings can contribute to the control of moisture transfer, gas exchange, and oxidation processes in fruits [65]. Edible coatings can be made of proteins, polysaccharides, and lipids. Among these materials, polysaccharides such as chitosan, alginate, carrageenan, and agar have been widely studied for the development of edible coatings. These coatings can also be used as carriers of different substances such as antimicrobials, nutraceuticals, and antioxidants. Table A2 summarizes different studies that evaluate the effect of these edible coatings alone or in combination with other functional substances in quality and safety of fresh blueberries.

Coatings made of chitosan have been extensively evaluated in blueberries. Chitosan, derived from the chitin polymer, is a good film former with a broad antimicrobial activity and compatibility with other substances [66]. Its antimicrobial action mechanisms include the alteration of cell permeability, interference with the synthesis of proteins, and the quelation of nutrients and oxygen for cells [67,68]. However, as disadvantage, it has been reported that chitosan has weak mechanical properties and limited barriers to water vapor [69]. Chitosan has shown better antifungal activity than coatings with sodium alginate. However, blueberries coated with sodium alginate have shown better firmness and lightness after 45 days of storage at 0 °C [63,70]. Jiang et al. [71] demonstrated that blueberries coated with 6 mg/mL chitosan showed better quality parameters after 35 days of storage at 2 °C compared to the control samples; the berries treated with low molecular weight chitosan showed a firmness of 159.1 g, while the control had a value of 117.0 g at the end of storage. Likewise, the total phenolic and anthocyanin values were 417.0 and 178.1 mg per 100 g of fresh weight, respectively, in the blueberries treated, and 382.8 and 141.3 mg per 100 g, respectively, in the control samples.

Other coatings have been used to increase the antimicrobial effect of chitosan and to extend the shelf-life of blueberries. Sun et al. [72] found that the incorporation of trans-cinnamaldehyde essential oil at a concentration of 0.5% to the chitosan coating provided an improved protective effect against softening and decreased the microbial population of blueberries after storage at 10 °C for 7 days, compared to the untreated berries and those coated with chitosan alone. Coatings made of a mixture of quinoa protein, chitosan, and sunflower oil delayed the ripening of blueberries and controlled mould growth for 32 days at 4 °C. However, the firmness and colour of the fruit were affected by the coating [73]. Another study used a coating based on chitosan, glycerol, Tween 80, and *Aloe vera* extract, which controlled fungal deterioration and prolonged the shelf-life of blueberries for 5 days [74]. Alvarez et al. [75] found that the addition of oligofructose and orange fibre to chitosan coatings increased the antifungal effect. Similarly, chitosan coatings enriched with inulin, oligofructose, and apple fibre extended the shelf-life of blueberries for 6 days. Yang et al. [76] showed a delay in decomposition and water loss in blueberries coated with chitosan plus 12% blueberry leaf extract during storage in a modified atmosphere (3 kPa O_2_ + 12 kPa CO_2_) at 2 °C, while total phenolic content and radical scavenging activity were preserved during storage.

In recent years, research efforts have been directed to the development and evaluation of coatings based on marine polysaccharides with antiviral potential. In this sense, coatings made of carrageenan and tea extract reduced the infectivity of MNV by more than 3.5 log in fresh blueberries kept at ambient temperature and between 2.4 and 3.1 log under refrigeration conditions. In the case of HAV, the effect of these coatings was more accentuated at 25 °C with reductions between 1.8–2.8 log. The coatings preserved the firmness and appearance of the blueberries [77]. On the other hand, the addition of *Larrea nitida* extract to the alginate/agar coating had a synergistic effect on the reduction of MNV in blueberries stored at 10 °C [78]. The above findings are an important basis for the development and optimization of formulations with the addition of other substances that allow—in addition to the reduction of pathogens—the deterioration caused by moulds to be minimized. Additionally, it would be pertinent to evaluate physicochemical parameters in blueberries during storage conditions.

Finally, liposomes as nano-carriers are presented as an alternative to obtaining an improved delivery of antimicrobial substances. In this sense, liposomes with 50 µM limonene reduced mould deterioration in blueberries by more than 60% at the end of nine weeks of storage at 4 °C [79].

All in all, edible coatings technology in combination with other substances is a natural alternative for preserving the quality and safety of fresh blueberries while providing an added value to the final product. However, the cost of materials, the loss of waxy bloom in the blueberries, and the difficulties encountered in the coating operations are current limitations to bear in mind. Furthermore, the effect of coatings should be evaluated in the different cultivars to avoid quality defects. The incorporation of nanotechnology in the application of antimicrobial components currently constitutes a promising alternative for further research.

## 4. Physical Strategies

Physical technologies are those achieving microbial decontamination through physical means, without the addition of chemical sanitizers or biopreservatives. However, many of these technologies are used in combination with other chemical methods, to increase their antimicrobial efficiency and better preserve the quality of food products.

Classically, there is a distinction between thermal and non-thermal technologies. Thermal technologies are based on the application of heating regimes for microbial decontamination of fruits and vegetables that are to be processed in the industry. However, they are not suitable for disinfection of fruits and vegetables intended to be consumed as fresh due to the degradation of their organoleptic characteristics. Instead, non-thermal technologies are widely used since they do not affect to any great extent the organoleptic and structural properties of fruits and vegetables with a minimal loss of heat-sensitive bioactive compounds [26,80].

Since most published studies of physical decontamination of blueberries rely on the use of non-thermal technologies, this section will describe the evaluation and optimization of such technologies for their use in blueberries and derived products (Table A3).

### 4.1. Ultraviolet Light

Ultraviolet (UV) light is an electromagnetic radiation with wavelengths between 100 and 400 nm [27]. According to the spectrum, it is classified as UV-A (315–400 nm), UV-B (280–315 nm), UV-C (200–280 nm), and vacuum UV (100–200 nm) [81]. It has been reported that UV-C has the highest antimicrobial effect; at 254 nm it inhibits the DNA synthesis of microorganisms, which leads to their inactivation [82].

However, application of UV-C alone does not achieve a substantial microbial reduction on berries. This is because microbial decontamination of fresh products by this technology is limited due to its low penetration capacity, sample heating, and shading effect [83]. Furthermore, at low UV doses, injured cells can be gradually recovered during storage. As regards the effect of UV on sensory quality, several studies have found no significant changes in the colour or texture of blueberries [84,85,86].

To overcome the abovementioned limitations of this technology, different alternatives have been proposed. One of them is the use of water-assisted UV systems (WUV). Among the advantages of these systems are the more uniform exposure of products to UV light, the synergistic action between UV and other chemical disinfectants added to water, and the inactivation of microorganisms in water once the products are washed [83]. Regarding this, several investigations have shown greater efficacy for blueberry decontamination in WUV compared to dry UV (Table A3). As an example, on a small scale with exposure times of 2 min WUV, treated blueberries showed a great reduction of MNV-1 (>4.2 log PFU/sample) compared to dry UV treatment (2.5 log PFU/sample) [83]. Additionally, the treatment was comparable to washing in 10 ppm chlorine for 2 min (>4.6 log PFU/sample). In the same study, the combination of WUV plus 10 ppm chlorine in a large-scale experiment had an enhanced inactivation compared to the independent technologies. This work also highlighted the difficulty of using UV in eliminating microorganisms present on rough surfaces such as the blueberry calyx, in which the reductions after all treatments were <2 log PFU/sample [83]. This result confirms what other researchers have reported—a greater reduction of pathogens on the skin than on the blueberry calyx after application of UV together with chemical methods [52,86,87]. On the other hand, Huang et al. [88] proposed WUV (23–28 mW/cm^2^) combined with PAA (80 ppm) for 2 min as recommended treatment for fresh blueberries due to its great efficiency in reducing *Salmonella* in water with elevated turbidity conditions. It is also noteworthy that the treatment achieved residual *Salmonella* cells in wash water below the detection limit. However, none of the treatments were able to eliminate *Salmonella* in the assayed berries completely.

Finally, photocatalysis technology has been studied as an alternative to overcome the limitations of UV light [89,90]. The microbial inactivation by UV-TiO_2_ photocatalysis is due to the production of hydroxyl radicals that are generated when the TiO_2_ is illuminated with UV light at wavelengths <385 nm [91]. In this sense, UV-TiO_2_ photocatalysis (4.5 mW/cm^2^) for 30 s achieved higher bacterial reductions (5.3 log CFU/g) on blueberry skin than applying UV alone at 6.0 mW/cm^2^ (4.5 log CFU/g). Both treatments increased the total phenol and anthocyanin content [91]. This study is in line with other findings showing the accumulation of bioactive compounds after exposure to UV light, which indicate a defence response of the fruits to the stress generated by the irradiation [92,93].

Thus, the use of UV light is conditional upon its combination with other disinfection technologies given its relatively low efficiency, especially when dealing with irregular shaped fruits, or in the presence of high organic material in water-assisted treatments.

### 4.2. Pulsed Light

Pulsed light (PL) is a physical technology where the inactivation of microorganisms is achieved using short time-high intensity light pulses. PL includes a wide wavelength range from UV to near infrared (200–1100 nm) [94]. Fluence is considered one of the most important factors in PL treatments [95]. A fluence level up to 12 J/cm^2^ is allowed for food applications [96]. Likewise, factors such as the distance from the light source to the sample or the food characteristics also influence the efficiency of the treatment [94,95]. One of the main benefits of PL is the achievement of significant microbial reductions in short exposure times [94]. In blueberries, a treatment of PL for 12 s at a fluence of 11.4 J/cm^2^ achieved log reductions of MNV-1, *Salmonella* and *E. coli* O157:H7 of 3.2, 3.8 and 5.6, respectively [97]. It has been reported that PL has a greater antimicrobial effect than UV light [35]. Nevertheless, implementation of PL equipment is more expensive than UV light and application of high fluences can also lead to deterioration of quality attributes due to the increase of product temperature [23,35]. It should also be noted that—similar to UV—PL treatments have limitations such as a low penetration in solid foods or a non-uniform exposure, among others.

To solve these inconveniences, several investigations have been focused on the use of wet PL (WPL) for microbial decontamination of blueberries and other derived products [23,98,99]. Huang et al. [23] demonstrated that dry PL for 60 s caused discoloration in blueberries which was related to the increase of temperature of the samples; in contrast, the WPL treatment helped to mitigate the heating effect while preserving the sensory quality of blueberries. Although the application of WPL showed a good performance against bacterial pathogens, YMC were lightly reduced, i.e., less than 1 log CFU/g.

Some authors [98] have studied the effect of WPL, showing, as expected, lower levels of *Salmonella* and YMC than untreated samples after storage at room temperature for 3 days and at 5 °C for 7 days. In addition, when comparing with dry PL (6 J/cm^2^) for 30 s and washing water, WPL (9 J/cm^2^) for 45 s was more efficient in the microbial decontamination of blueberries. In terms of quality, WPL seemed not to produce substantial changes in the decontaminated product, finding that WPL did not affect the total phenolic content and minimally impacted the colour of blueberries. In contrast, anthocyanin content and antioxidant activity were slightly lower in WPL compared to dry PL. At room temperature, the authors noted the undesired presence of leaks in blueberries treated with water and WPL, limiting the use of water for subsequent non-refrigerated storage.

Other studies evaluating the use of UV and PL technologies have shown that they can be equally effective against pathogenic bacteria when comparing the performance between WUV and WPL [100]. The studies found that there were no significant differences in the reduction of *Salmonella* in blueberries. Despite the advantages of the water-assisted system, the residual moisture resulting from the process can favour the deterioration of blueberries for which it is advisable to apply drying to reduce alteration during storage.

Concerning PL technology, future efforts should be focused on improving the processing conditions and obtaining more economical equipment for its implementation on an industrial scale.

### 4.3. High Hydrostatic Pressure

High hydrostatic pressure (HHP) is a consolidated non-thermal technology in which food is generally exposed to pressures of 300–600 MPa conveyed by water. High pressure causes microbial death due to the destruction of the cell membrane and wall, and also affects proteins [80]. The antimicrobial effect of this method depends on factors such as temperature, pH, treatment time, product characteristics, and pressure resistance of microorganisms [101].

Published studies on blueberries showed that human norovirus and its surrogates were more sensitive to pressure in wet than in dry blueberries, and the effectiveness of HHP inactivation increased when the sample temperature decreased (Table A3). Other authors found that a treatment of 550 MPa for 2 min at 0 °C achieved a satisfactory inactivation of HuNoV GI.1 and GII.4 on fresh blueberries and in puree [102]. Furthermore, the quality attributes of the puree (general appearance, viscosity, aroma, colour, and general acceptability) were not adversely affected by the treatment, in contrast with the textural modifications induced by HHP in fresh fruits. As regards textural modifications, other researchers showed that blueberries were softened during pressurization and concluded that HHP intended for fresh blueberries was inadequate [103,104].

The synergy of natural antimicrobials and HHP is presented as another alternative to increase the efficiency of this technology. In this regard, Kabir et al. [105] evaluated the addition of carvacrol and caprylic acid for the inactivation of serogroups O157 and non-STEC *Escherichia coli* in blueberry juice. As an example, while at 450 MPa and 4 °C, the D-value for non-STEC was 8.03 min, when 0.5% carvacrol was added, the D-value was reduced to 2.92 min [105]. Additional factors such as pathogen adaptation to food matrix or selection of strains according to their pressure resistance should be considered when evaluating the efficiency of this technology.

It can be concluded that HHP constitutes an effective technology mainly for the treatment of blueberry derivatives with minimal effects on quality.

### 4.4. Pulsed Electric Field

Pulsed electric field (PEF) is a technology based on the application of electric pulses of short duration (μs) and high voltage (20–80 kV/cm) to foods placed between two electrodes [106]. This process produces a rupture of the cell membrane, and thus the inactivation of microorganisms [107]. Among the factors that determine the lethal effect of PEF are electric field strength, treatment time, pulse shape, and start temperature. Similarly, the nature of microorganisms and product parameters such as composition, pH, or conductivity, among others, are also considered as factors influencing the extent of microbial inactivation by PEF [108]. However, limitations of this technology are its high cost and technical difficulties in setting the parameters’ targets [109].

The use of combined strategies using PEF and other disinfectants is reported in the literature. The combination of PEF plus PAA (0.25%) for 4 min allowed efficient decontamination of blueberries compared to the use of PEF alone (2 kV/cm). As an example, *E. coli* was reduced by less than 0.5 log CFU/g with the PEF treatment, while the combined treatment achieved up to 2.9 log CFU/g reduction [110]. In that study, long treatment times were used to compensate for low field strength. Although the combined treatment preserved the colour and increased the concentration of anthocyanins and phenolic compounds by 10% and 25%, respectively, the texture of the fruit was damaged, indicating that the treatment is not recommended for fresh blueberries.

In liquid matrices, Chen et al. [111] evidenced an increase in the inactivation of *E. coli* in blueberry juice by increasing the electric field and processing time. In this sense, the treatment at 35 kV/cm and 82 μs reduced the pathogen by 5.12 log CFU/mL. Regarding the quality of the juice, PEF treatment at 30 kV/cm, 54 μs, and 1.4 ms/cm maintained colour, aroma, and greater retention of vitamin C and anthocyanins after storage at 4 °C for 30 days compared to heat treatment while achieving commercial sterility. Other parameters such as acidity and phenol content changed slightly after treatment [111].

In order to overcome the cost and to control the settings of parameters of the PEF equipment, microchips have been designed and improved for low-voltage PEF sterilization [109]. This technology has shown great potential in microbial inactivation while preserving the quality of juice. For example, treatment with microchip-PEF at 400 V and 0.2 ms efficiently inactivated spoilage microorganisms in blueberry juice [112]. Likewise, blueberry juice treated with microchip-PEF (350 V, 0.15 ms, 7 mL/min) was microbiologically stable and preserved its colour and nutritional properties after storage for 30 days at 4 °C [113].

In the light of the published studies, PEF seems to be a valid alternative for microbial disinfection of derived blueberry products being able to preserve most of the quality parameters. However, its use on fresh blueberries should be optimized because of damage to the texture of the blueberries.

### 4.5. Cold Plasma

Cold plasma (CP) is a novel non-thermal technology based on the use of ionized gases [33]. CP is generated from air or other feed gases by different energy sources [114]. The microbial inactivation by this technology is achieved by UV light and reactive chemical products derived from the ionization process [114]. However, it has been reported that this technology could produce some undesirable organoleptic changes in blueberries, such as a reduction of firmness and anthocyanin content and changes in colour parameters [115].

CP has been studied by different researchers for disinfection of blueberries, as shown in Table A3. Though optimization of this technology is required for its use in blueberries, it was shown to be effective in reducing microbial contamination of norovirus surrogates, as demonstrated by Lacombe et al. [116]. In relation to the native microbiota, CP has been shown to be more effective in the inactivation of bacteria than fungi [115,117,118]. In this way, Pathak et al. [118] recommended nitrogen-generated CP treatments greater than 5 min for effective reduction of moulds and yeasts.

In relation to the effect of CP on the sensory and nutritional aspects of blueberries, the different findings can be attributed to the different treatment conditions. Lacombe et al. [115] associated the reduction of firmness and anthocyanin content in blueberries treated with air-generated CP (air plasma jet, 47 kHz and power consumption of 549 W) to the increase in temperature and the presence of ozone and other free radicals. Softening of the fruits was also attributed to mechanical damage during the process. Similarly, the colour was significantly affected after 120 s for *L* * and *a* * values, and 45 s for *b* *. In this sense, blueberries became darker and with an increase of the perceived surface red and blue colours. On the contrary, Dong et al. [117] reported an increase in the contents of sugar, vitamin C, and total anthocyanin in blueberries treated with air-generated CP (dielectric barrier discharge with 19.7 kHz, and 6.48-W input power consumption) and stored for 20 days in comparison with control samples. Pathak et al. [118] did not find significant differences regarding the content of ascorbic acid, anthocyanins, solids, and titratable acidity in blueberries treated with N-generated CP (diffuse coplanar surface barrier discharge, 300 V) and stored for 10 days at 7 °C with respect to control fruits. However, the authors recommended treatments of less than 10 min to avoid significant changes in texture.

Regarding the application in blueberry juice, Hou et al. [119] found that the application of CP achieved an increase in the content of phenolics and preserved the original colour compared to the heat treatment. In contrast, treatment times of less than 4 min were suggested to maintain the anthocyanin content and vitamin C in the samples.

In comparison with other strategies, high antimicrobial activity of CP against *B. cinerea* was demonstrated by Zhou et al. [120] on blueberries *versus* the use of aqueous ozone after storage at 20 °C for 8 days. The blueberries treated with CP showed a high firmness and ascorbic acid concentration.

In summary, CP is a technology with great potential for microbial decontamination of blueberries. However, the optimization and standardization of process parameters (power, plasma source, setup, and treatment time) are necessary. In addition, further inactivation studies evaluating the effect of technology on microbial fate and product quality under different storage conditions is required.

### 4.6. Ionizing Irradiation

Ionizing irradiation, such as *X*-rays, gamma rays and electron beams act on water molecules forming free radicals that inactivate microorganisms [33]. However, softening and quality loss of fruits treated at high doses is one of the limitations of this technology [121]. The FDA restricts the maximum level of irradiation of fresh fruits and vegetables to 1.0 kGy [122].

The antimicrobial effect and preservation of quality attributes after an irradiation treatment in blueberries can differ according to published studies. Doses of gamma irradiation of 0.4 kGy had a limited effect on the population of moulds and yeasts and pathogenic bacteria in blueberries during storage at 0–1 °C for 31 days [123], being 11% softer than control samples, while soluble solids content, titratable acidity, and weight loss were not affected by the treatment. However, other authors [124] found that *Toxoplasma gondii* oocysts were reduced by 4 log PFU/g at low doses of gamma irradiation (0.2, 0.4 and 0.6 kGy at 4 °C) without affecting firmness, anthocyanins, and colour in blueberries. Shelf-life studies should be performed to evaluate the effect of processing parameters during the storage period.

Regarding the nature of rays, although the electron beam has less penetration ability compared to gamma rays, electron beam irradiators have the advantage of being electronic in nature, meaning a potential alternative as safety concerns are not comparable to cobalt-60 concerns, the latter being implicated in gamma rays technology. Likewise, electron beam irradiation allows the use of high doses with great precision [125,126,127]. Kong et al. [125] showed that electron beam irradiation at low doses (<3 kGy) extended the shelf-life of blueberries without affecting their antioxidant activity, total monomeric anthocyanin, and L-ascorbic acid content. The same study found a D_10_ value average for *E. coli* K-12 of 0.37 kGy on blueberries. On the other hand, Nambeesan et al. [127] reported that blueberry cultivars treated with doses of 1.0 kGy showed reductions of ≤1 log for the total of aerobic bacteria and yeasts after 6 days at 2–4 °C. In the case of coliforms, the reductions were <1 log for the “Farthing” cultivar and 2 log units for “Rebel”. Likewise, the authors did not find any significant effect of irradiation on the incidence of post-harvest diseases, concluding that higher doses would be required for the elimination of plant-pathogenic fungi in blueberries. The firmness was only reduced in the “Farthing” cultivar. Other parameters such as soluble solids content or titratable acidity were not affected.

All in all, at the permitted doses, irradiation helps to reduce the microbial load but does not guarantee neither the safety of blueberries nor spoilage control. The combination of irradiation with other technologies could be considered in future investigations to achieve greater efficiency for the disinfection of fresh blueberries.

### 4.7. Ultrasound

Ultrasound (US) is considered another technology useful for the treatment of fruit and derivatives. The antimicrobial effect of US is attributed to cavitation and the formation of free radicals which alter the cell wall and membrane producing damage in DNA structure [127]. The efficiency of this method depends on several factors including wave frequency, power, and exposure time [128]. Likewise, this technology needs to be accompanied by other chemical or physical strategies due to its limited antimicrobial effect [33,35].

To increase the efficiency of US, it can be used together with pressure treatment (manosonication), heat treatment (thermosonication), or both (manothermosonication). In this sense, moulds and yeasts were completely inactivated in nectar and blueberry juice with high power ultrasound (20 kHz) in combination with heat (60 °C) and treatment times of 3–9 min [129]. Zhu et al. [130] found that manothermosonication (560 W, 40 °C/350 MPa, 40 °C) achieved a rapid inactivation of *E. coli* O157:H7 in blueberry juice after 5 min, while 97.49% of anthocyanin content was retained and the polyphenol oxidase activity decreased to 10.91%. In relation to the treatment of fresh products, the combination of US with natural chemicals has been explored as an alternative to improve the safety and efficacy of sanitation processes (Table A3). However, the long treatment times make its industrial application unfeasible. More studies are required to up-scale this technology [131].

## 5. Biological Strategies

Finally, biological methods are considered as a promising alternative to improve the quality and safety of fresh fruits [33]. Treatments based on the use of bacteriophages, lactic bacteria, metabolites of microorganisms, and other compounds of biological origin have been tested for the reduction of pathogen and spoilage in vegetables [27,132]. Moreover, in recent years, several studies have evaluated biological strategies as alternatives to chemical and physical methods for microbial decontamination of blueberries. For example, washing with 1% denatured lysozyme for 1 min was comparable to washing with 100 ppm chlorine, and it showed reductions of HAV and MNV-1 of 3.9 log MNP/g and 4.2 log PFU/g, respectively, in fresh blueberries [133]. Likewise, Bambace et al. [134] evaluated the application of alginate coatings with the addition of fructo-oligosaccharides and probiotic lactobacilli in fresh blueberries. The coating was able to inhibit *L*. *innocua* counts by 1.7 log and the firmness and sensory acceptability of blueberries was unaffected for 14 days in refrigeration, time after which the highest rate of fungal decay was observed (60% at 21 days). On the other hand, the inclusion of cyclolipopeptides produced by *Bacillus subtilis* to alginate coatings decreased respiration and fungal contamination in blueberries stored at −1 to 0 °C for 20 days [135]. On the other hand, the use of different biological compounds such as vegetable extracts and essential oils has been widely evaluated in blueberries (Section 3.5). The use of pullulan coatings, an exopolysaccharide produced by the fungus *Aureobasidium pullulans*, combined with propolis extract reduced the population of bacteria and moulds by 3–4.5 log in fresh blueberries stored at 16 °C for 21 days. In addition, these coatings contributed to the delay in the ripening of blueberries and the decrease in water loss [136]. The application of allyl isothiocyanate, a natural compound present in plants of the *Cruciferae* family, reduced blueberry decomposition during storage at 10 °C for 21 days, which was associated with the generation of reactive oxygen species. However, the contents of total phenolics, anthocyanins, and antioxidant capacity of blueberries decreased [19]. Finally, the use of hexanal, a natural plant volatile, has shown antifungal properties in blueberries. In this sense, three applications of 900 ppm of hexanal vapor for 24 h at 0.5 °C combined with controlled atmosphere storage (10–12 kPa O_2_ and 12–15 kPa CO_2_), allowed for a lesser decay and longer shelf-life after 15 weeks of storage at 0.5 °C [137].

Biological strategies constitute a natural alternative to promote the safety and quality of blueberries destined for the fresh market, although not enough studies have yet been conducted. The development of blueberries with multifunctional properties (e.g. probiotics-added blueberries) is an important research line where substantial efforts should be invested.

## 6. Conclusions

In the last decades, different chemical and physical strategies have been evaluated for microbial decontamination of blueberries. It is only recently that biological methods have been tested. However, there are future perspectives for this kind of applications in blueberries. Several of these strategies showed comparable results and were even superior to traditional disinfection methods, and in addition, they greatly overcame side effects observed with the use of classical disinfectants such as chlorine.

To summarise, according to the studies reviewed, PL and CP technologies show promising results for use in blueberry decontamination. Among the chemical strategies, PAA, EOW, and aqueous ozone have great potential to replace chlorine for the elimination of microorganisms on the surface of blueberries. Gaseous chlorine dioxide and edible coatings are advantageous for blueberries intended for the fresh market. In relation to derived products such as blueberry puree or juice, technologies such as HHP and PEF achieve satisfactory microbial inactivation with minimal impact on the sensory and nutritional quality of the products. Likewise, it is a common observation that the use of combined technologies by application of the well-known “hurdle technology” offers greater antimicrobial effectiveness while preserving the quality of the products [80]. Further studies should evaluate the potential of technologies against microbial adhesion on the surface of blueberries since most of the published studies have carried out disinfection treatments within a few hours after inoculation. Future inactivation studies should also cover a broader range of pathogens such as viruses or parasites, including the effect of variability between strains.

Finally, research efforts should be focused on the effect of these strategies on microbial inactivation as well as on the sensory and nutritional quality of blueberries, especially during their shelf-life. It is only with this approach that an integral evaluation of these technologies can be performed.

## Abbreviations

CFU	colony-forming units
COD	chemical oxygen demand
CP	cold plasma
EOW	electrolyzed oxidizing water
FCV	feline calcivirus
FDA	Food and Drug Administration
HAV	hepatitis A virus
HHP	high hydrostatic pressure
HuNoV	human norovirus
MNV	murine norovirus
MS	manosonication
MT	manothermal
MTS	manothermosonication
NTU	nephelometric turbidity unit
PAA	peracetic acid
PEF	pulsed electric field
PFU	plaque forming units
PL	pulsed light
SDS	sodiumdodecyl sulfate
TAB	total aerobic bacteria
TPC	total plate count
TS	thermosonication
TV	tulane virus
US	ultrasound
UV	ultraviolet light
WPL	water-assisted pulsed light
WUV	water-assisted UV
YMC	yeasts and moulds count.

## Appendix A

**Table A1:** Main advantages and limitations of strategies for the control of microorganisms on fresh blueberries and products derived thereof.

Strategies	Advantages	Limitations	Reference
Chlorine dioxide (ClO_2_)	Gaseous chlorine dioxide (gClO_2_) - High penetration ability - Products do not require a subsequent washing - Greater efficacy than chlorine - Wide range of antimicrobial action Aqueous ClO_2_ - Higher antimicrobial efficacy at neutral pH than chlorine - Fewer carcinogenic halogenated disinfection by-products (DBP) than chlorine	Gaseous gClO_2_ - Possible bleaching of fruits - Antimicrobial activity affected by gas concentration, time of exposure, humidity and temperature Aqueous ClO_2_ - Low efficiency at permitted concentrations - Products require washing after treatment	[31,33,35]
Ozone (O_3_)	- Generally recognized as safe (GRAS) - Decomposes to nontoxic products - High microbial action against bacterial pathogens	- Risk of explosion and toxicity - At high concentrations it can cause undesirable organoleptic changes - Requires on-site generation - High concentrations and exposure times are required to achieve significant microbial reductions - Less effective against moulds and yeasts	[33,45,49]
Peracetic acid (PAA)	- Environmentally friendly - Tolerant to pH, temperature, and organic load - Effective against bacterial pathogens	- Requires higher concentrations than those allowed (80 ppm) to achieve significant microbial reduction	[33,53]
Hydrogen peroxide (H_2_O_2_)	- Use in aqueous and gaseous form - No residue production	- High concentrations can affect product quality	[33,58]
Organic acids	- No toxicity - Economical and easy to use	- Requires long exposure times to achieve significant microbial reduction - Interferes with sensory quality	[33]
Edible coatings	- Antimicrobial action depending on the type of material used - Can be used as carriers of different substances	- High cost of materials - Loss of waxy bloom in the blueberries - Drawbacks in coating operations	[45,66]
Ultraviolet (UV)	- Economical equipment and easy to use - UV exposure can induce the synthesis of bioactive compounds such as anthocyanins - UV treatment can be combined with water (water-assisted UV systems) in overhead and submersible settings	- Low penetration capacity - Sample heating - Shading effect	[33,83]
Pulsed light (PL)	- Significant microbial reductions in short exposure times - Balanced cost - PL can be combined with water (water-assisted PL systems)	- Low penetration in solid foods - High fluences can also lead to deterioration of quality attributes	[35,94]
High hydrostatic pressure (HHP)	- Microbial and enzymatic inactivation - No degradation of flavour and nutrients	- Expensive equipment - Antimicrobial action depends on the type of microorganism, processing factors and product parameters - Not suitable for fresh blueberries	[33]
Pulsed electric field (PEF)	- Treatment of liquid matrices - Minimal impact on product quality	- Antimicrobial action influenced by product parameters and type of microorganisms - Not suitable for fresh blueberries - High cost	[109,110]
Cold plasma (CP)	- Can be used in liquid and solid products - Could be applied to the packed product	- Possible changes in sensory properties and in the content of bioactive compounds - Limited effect against moulds	[33,116]
Ionizing irradiation	- Can be used at room temperature and after packaging	- High doses affect the texture and quality of the product - Limited effect against spoilage microorganisms	[33,122]
Ultrasound (US)	- Can be used in both liquid and solid matrices - Minimal impact on the quality of derived products	- Needs to be combined with other strategies to be more effective	[33,35]
Biological compounds	- Natural alternative - Added value to products	- Some compounds such as essential oils can affect the organoleptic characteristics of the products - More studies are required	[27,33]

**Table A2:** Chemical strategies for the control of microorganisms on fresh blueberries.

Chemical Disinfectants	Microorganisms	Treatment Conditions	Treatment Efficiency (log Reduction)	Reference
Gaseous chlorine dioxide (gClO_2_)	TAB, YMC, *E. coli, C. acutatum*	0.3–0.35 gClO_2_ per pad	TAB and YMC: Storage at 20 °C: ≥1.3 log CFU/g Storage at 10 °C for 8 d: ≥1.7 log CFU/g Storage at 10 °C: *E. coli*: 2.2–3.3 log CFU/g *C. acutatum*: 1.3–2.0 log CFU/g	[40]
gClO_2_	Shiga toxin-producing *E*. *coli* (STEC), *Salmonella*, and *L*. *monocytogenes*	Cumulative gClO_2_ at 1529 ppm-h	STEC, *Salmonella* and *L*. *monocytogenes*, respectively: 3.9, 3.9, and 4.6 log CFU/g.	[37]
gClO_2_	TV	gClO_2_ produced by acidified NaClO_2_ solutions ranging from 0.1–10 mg. Treatment times: 5–330 min	gClO_2_ (0.1 mg NaClO_2_): 1 log after exposure for 30–330 min gClO_2_ (1 mg NaClO_2_): 2.2 log after 15 min	[39]
gClO_2_	TV	0.63–4.40 (ppm-h/g product)	0.63 (ppm-h/g): 3.82 log PFU/g	[38]
gClO_2_	*S*. *enterica* serovars	3.55–6 (ppm/h/g product)	5.5 (ppm/h/g): 5.63-log CFU/g	[32]
gClO_2_ and Ozone (O_3_)	STEC, *S. enterica*, *L. monocytogenes*	gClO_2_: 0.04, 0.07, 0.15 mg ClO_2_/g produce for a 5.0 h exposure.	gClO_2_ was the best treatment: 3.7, 2.7, and 2.1 log CFU/g in STEC, *Salmonella* and *L. monocytogenes,* respectively (0.15 mg for 5 h).	[51]
Sodium hypochlorite (NaClO), ClO_2_ and Peracetic acid (PAA)	MNV-3	NaClO: 50 ppm ClO_2_ 20 ppm PAA: 85 ppm Treatment time: 1 min	With organic matter simulation: NaClO: >4 log PFU/mL PAA: ∼3 log PFU/mL	[42]
ClO_2_	TAB and YMC	100 ppm for 10 min	TAB: 1.4–1.5 log CFU/g. YMC: 0.8–0.9 log CFU/g.	[41]
Chlorine and O_3_	TPC and YMC	Chlorine spray: 100 mg/L. Aqueous ozone sprays: 1 mg/L. Treatment time: 60 s.	Chlorine was the most effective treatment after 12 months at −18 °C. TAB: below the detection limit (20 CFU/g). YMC: 1.5 log CFU/g (yeasts) and 2 log CFU/g (moulds).	[138]
Electrolyzed oxidizing water (EOW), Ultraviolet light (UV), O_3_ and O_3_ + UV	*E. coli* O157:H7	EOW: 46.1 mg/L of residual chlorine for 4–26 s. UV: 20 mW/cm^2^ for 1–10 min. O_3_: 4000 mg/L for 1 min O_3_ + UV: 7.95 mW/cm^2^ for 2 min + 4000 mg/L for 1 min.	On calyx and skin of blueberries, respectively: EOW: 0.13–0.24 and 0.88–1.10 log CFU/g. O_3_: 1.02 and 1.64 log CFU/g UV/10 min: 2.14 log and 4.05 log CFU/g. O_3_ + UV: >1–2 log CFU/g in calyx of blueberries than single combinations.	[52]
EOW, Bleach solution, ozonated water and FIT^®^ solution	*E. coli* O157:H7	EOW: 30 mg/L free chlorine. Bleach solution: 100 mg/L free chlorine. Ozonated water: 1.5 mg/L O_3_. FIT^®^ solution (levulinic acid). Treatment time: 1–5 min	Bleach solution: 4.4–4.8 log CFU/g. EOW: 3.9–4.4 log CFU/g. FIT solution: 3.3–4.6 log CFU/g. Ozonated water: 2.3–3.5 log CFU/g.	[49]
Controlled atmosphere storage or O_3_	*L. monocytogenes*	Controlled atmosphere storage: 5% O_2_, 15% CO_2_, 80% N_2_. Ozone gas: 4 ppm at 4 °C or 2.5 ppm at 12 °C. Treatment time: 10 days at 4 °C or 12 °C	Ozone gas was the most effective treatment: 3 and 2 log CFU/mL at 4 and 12 °C, respectively.	[50]
PAA, acidic EOW, near neutral EOW, bleach and lactic acid.	Strains of *E. coli* O157:H7, *S.* Typhimurium DT104, and *L. monocytogenes*.	PAA: 45–100 mg/L. Acidic EOW, near neutral EOW, and bleach: 100 mg/L free chlorine. Lactic acid: 2%. Treatment time: 5 min	PAA at 100 mg/L was the best treatment. *E. coli* O157:H7 6.7 log CFU/g. *S.* Typhimurium DT104 5.9 log CFU/g.	[55]
Chlorine and PAA	*L. monocytogenes* strains, TPC and YMC	Chlorine: 100 ppm. 0.4% Neo-Pure (PAA 450 ppm). Treatments with sanitizers for 2 min combined with storage at 4 °C (14 d) and at ×15 °C (28 d).	Nero-Pure treatments were more efficient than chlorine. Dip-inoculated berries: ∼0.9 log CFU/g of *L. monocytogenes* (after 14 d at 4 °C) ∼3.0 log CFU/g reduction during 28 d at −15 °C Spot-inoculated berries (4 °C during 14 d): 8.2 log CFU/g at high inoculation level Spot-inoculated berries (−15 °C during 14 d): Enrichment negative TPC and YMC: ∼0.8 and 1.6 log CFU/g, respectively, after 24 h of cold storage.	[56]
Sodiumdodecyl sulfate (SDS) in combination with antimicrobial agents.	*S. enterica* serovar Typhimurium and YMC	Chlorine: 4–200 ppm. Lactic acid, acetic acid, and citric acid: 0.05 mg/mL or 0.5 mg/mL. H_2_O_2_: 50–200 ppm. All treatments combined or not with SDS: 50–500 ppm. Treatment time: 1 or 5 min.	0.5 mg/mL acetic acid + 5000 ppm SDS (5 min): *Salmonella*: 4.0 log CFU/g. YMC: 2.4 log CFU/g 200 ppm hydrogen peroxide + 5000 ppm SDS (5 min): *Salmonella*: 4.2 log CFU/g. YMC: 2.0 log CFU/g.	[61]
Electro-activated solutions of weak organic acid salts	*L. monocytogenes*. *E. coli* O157:H7, *Alternaria alternata, Fusarium oxysporum,* and *B. cinerea.*	Electro-activated solutions of 3% (*w*/*v*): potassium acetate, potassium citrate, and calcium L-lactate. Treatment time: 0.5–5 min.	The reduction was dependent on the treatment time. All solutions (5 min): L. monocytogenes: ~4 log CFU/g. Potassium acetate and potassium citrate solutions (5 min): *E. coli* O157:H7: ~3.5 log CFU/g. Potassium acetate solution (5 min): *A. alternata:* >3 log CFU/g *F. oxysporum* and *B. cinerea:* >5 log CFU/g	[62]
Limonene and liposomal nanoparticles with limonene.	YMC	50 µM limonene and liposomal nanoparticles with 50 µM limonene	Liposomes: >60% reduction of deterioration at the end of 9 weeks at 4 °C.	[79]
Hot water	TPC and YMC	Hot water: 60–90 °C with (0.05 and 0.1%) or without Boxyl ^®^ (atomic oxygen). Treatment time: 10–30 s.	TAB reduction increased with temperature. YMC reduction increased with temperature and contact time. 65 to 70 °C (10 to 15 s): TAB: 1.5 log CFU/g. YMC: 2.0 log CFU/g.	[139]
Edible coatings	TAB and YMC	Coating with 1% chitosan, 1% acetic acid, 0.75% glycerol, 0.25% Tween-20, and 0.1% to 0.5% essential oils (carvacrol, cinnamaldehyde, and trans-cinnamaldehyde).	Chitosan + 0.5% trans-cinnamaldehyde: TAB and YMC: 2–3 log CFU/g at 7 d at 10 °C.	[72]
Edible coatings	YMC	Quinoa protein (0.62% *w*/*v*), sunflower oil (3.8% *w*/*v*), and chitosan (2% *w*/*v*) in a ratio of 1:1 (*v*/*v*).	Coating delayed YMC during 32 d at 4 °C Control samples showed an increase of YMC (1.8–3.1 log CFU/g) between 20 and 35 d of storage at 4 °C.	[73]
Edible coatings	YMC	2% chitosan coating. 1.5% sodium alginate coating. 1.5% chitosan and 1% sodium alginate coating	After 45 d at 0 °C: Chitosan coating (both cultivars): Yeasts: 2.67–3.92 log CFU/g Chitosan coating (cv Berkeley): Moulds: 1.35 log CFU/g Alginate coating (cv O’Neal): Moulds: 2.74 log CFU/g	[63]
Edible coatings	*Botrytis cinerea* artificially inoculated and YMC	Chitosan or chitosan coating + *Aloe vera*. 0.5% (*w*/*v*) chitosan, 0.5% (*v*/*v*) glycerol, 0.1% (*w*/*v*) Tween 80, and 0.5% (*v*/*v*) *Aloe vera* liquid fraction.	After 25 days at 5 °C: YMC in non-inoculated blueberries: Chitosan coating: >1 log CFU/g Chitosan coating + *Aloe vera*: >2 log CFU/g *B. cinerea* in inoculated blueberries: Chitosan coating + *Aloe vera*: >1 log CFU/g	[74]
Edible coatings	YMC	2% chitosan coating. 1.5% sodium alginate coating. 1.5% chitosan and 1% sodium alginate coating.	After 45 days at 0 °C: Chitosan edible coating was the best treatment: Yeasts: >2.5 log CFU/g Moulds: >1 log CFU/g	[70]

**Table A3:** Physical technologies used alone or in combination with antimicrobial agents for the control of microorganisms on blueberries and products derived thereof.

Technology	Microorganisms	Food Matrix	Treatment Conditions	Treatment Efficiency (log Reduction)	Reference
Water-assisted UV (WUV)	MNV-1	Fresh blueberries	UV dose: 12.000 J/m^2^. Treatment time: 1–5 min. Treatments under different conditions of organic load.	Small scale clear water/2 min: WUV: >4.32 log PFU/sample UV: 2.48 log PFU/sample Large-scale water with COD 2150 mg/L (2 min): WUV + 10 ppm chlorine: 2.88 log PFU/skin sample WUV + 10 ppm chlorine: <2 log PFU/calyx sample	[83]
WUV	Strains of *E. coli* O157:H7 and *Salmonella enterica*	Fresh blueberries	UV: 7.9 mW/cm^2^ WUV: 4.6 mW/cm^2^ Treatment time: 2–10 min. 100 ppm of SDS, chlorinated water (10 ppm free chlorine), or 0.5% levulinic acid for 10 min with or without WUV (7.9 W/cm^2^).	WUV treatment > efficiency than UV (average >1.4 log CFU/g for spot-inoculated blueberries). UV treatments < efficiency (<2 log CFU/g for dip inoculated blueberries). Non-significant effect of added chemicals in dip-inoculated on blueberries.	[87]
WUV	*Salmonella enterica* strains	Fresh blueberry	UV in turbid tap water with or without 10 ppm free chlorine, 80 ppm PAA and 1% or 2% H_2_O_2_. UV intensity: 23 and 28 mW/cm^2^. Treatment time: 2 min	WUV-Chlorine and WUV-PAA were the best treatments. Large-scale study WUV-PAA and WUV-chlorine: 2.22 log CFU/g	[88]
WUV	*Salmonella enterica* strains	Blueberries	Submersible intensity: 4 mW/cm^2^ Treatment time: 2 min	1.8–2.0 log CFU/g	[85]
WUV	*Salmonella enterica* strains	Blueberries	Larger-scale study: Intensity WUV: 2–29 mW/cm^2^ Treatment time: 1 or 10 min	10-min WUV (29 mW/cm^2^) Spot and Dip inoculated products: 4.54–1.94 log CFU/g.	[86]
UV	*E. coli* O157:H7, serovars of *Salmonella enterica*, *L. monocytogenes*, *Enterococcus faecium*, *E. coli* P1, *Listeria innocua*, MS2 bacteriophage HAV, and MNV.	Fresh and frozen blueberries.	20 s (212 ± 25 mJ/cm^2^) 60 s (650 ± 71 mJ/cm^2^) 120 s (1331 ± 103 mJ/cm^2^)	HAV and MNV: >2–3 log/g in blueberries MNV in fresh berries > inactivation than in frozen berries, unlike HAV. *Salmonella*, *E. coli* O157:H7 and *L. monocytogenes*: <1 log/g Treatment time has not significantly improved inactivation in most of the matrices.	[84]
UV-TiO_2_ photocatalysis	*Escherichia coli* K12	Fresh blueberries	UV-TiO_2_ photocatalysis: 4.5 mW/cm^2^. UV: 6.0 mW/cm^2^. Treatment time: 0–10 min	UV-TiO_2_ photocatalysis > efficiency UV. 5.3 log CFU/g on the skin-inoculated blueberry (30 s). 5.2 log CFU/g on the blueberry calyx (10 min).	[91]
405-nm monochromatic blue light	TV	Fresh blueberries	Intensity: 4.2 mW/cm^2^. Treatment time: 5–30 min	Treatment times resulted in a reduction of <0.2 log PFU/mL. 1.01 log PFU/mL after a 405 nm light treatment + Rose Bengal.	[140]
Water-assisted pulsed light (WPL)	YMC, strains of *E. coli* O157:H7 and *Salmonella enterica.*	Fresh blueberries	Intensity: 5.0–56.1 J/cm^2^ Treatment time: 5–60 s	The inactivation was time-dependent *E. coli* O157:H7: 3.0 and >5.8 log CFU/g on blueberry calyx and skin, respectively (60 s). *Salmonella*: 3.6 and >5.9 log CFU/g on blueberry calyx and skin, respectively (60 s). WPL achieved a limited reduction of YMC.	[23]
WPL	Strains of *Salmonella enterica.*	Fresh blueberries	PL combined or not with 1% H_2_O_2_ and under different organic loading conditions. High PL: 0.225–0.298 J/cm^2^-pulse. Low PL: 0.102–0.140 J/cm^2^-pulse. Treatment time: 0.5–1 min.	No significant differences between the low and high creep PL treatments. WPL + 1% H_2_O_2_ (1 min) > efficiency for reducing *Salmonella* in clear water by >5.6 log CFU/g. The high organic load condition did not affect the efficacy of the WPL-H_2_O_2_ for 1 min.	[99]
WPL	*Salmonella enterica* serotypes and YMC	Fresh blueberries	PL dose: 6 J/cm2 (30 s). WPL dose: 9 J/cm^2^ (45 s).	WPL was the most effective treatment: *Salmonella*: >4.4 and 0.8 log CFU/g for spot and dip inoculation, respectively. YMC: 0.9 log CFU/g.	[98]
Pulsed light (PL)	MNV-1, *Escherichia coli* O157:H7 and *Salmonella* Newport	Fresh blueberries	Intensity: 5.9–22.5 J/cm^2^ Treatment time: 6–24 s.	Increasing PL fluence yielded significantly higher reductions for *Salmonella*. MNV-1: 3.1–3.8 PFU/sample *E. coli* O157:H7: 5.7 log CFU/sample (24 s) *Salmonella*: 4.2 log CFU/sample (24 s)	[97]
WPL and WUV	Strains of *Salmonella*	Blueberry	WUV: ~13 or 28 mW/cm^2^. WPL: ~0.15 or 0.30 J/cm^2^ per pulse. Treatment time: 1 or 2 min	Spot-inoculated: 4.5–5.7 log CFU/g Dip-inoculated: 1.8–2.3 log CFU/g	[100]
High hydrostatic pressure (HHP)	HuNoV GI.1	Fresh blueberries	Intensity: 400–600 MPa (wet-state samples) and 600 MPa (dry-state samples). Pressure treatments: 1 or 21 °C for 2 min.	Pressure inactivation was more effective at lower temperature and when blueberries were surrounded by water. 2.7 log (500 MPa at 1 °C, wet state) 0.5 log (500 MPa at 21 °C, wet state)	[103]
HHP	TV and MNV-1	Fresh blueberries	Intensity: 250–600 MPa (4, 21 or 35 °C). Treatment time: 2 min. Specific pressure units according to virus and sample.	Dry state blueberries: 400 and 600 MPa (3 temperatures): < 1 log PFU/blueberry. Pressure inactivation increased as sample temperature decreased. Wet state blueberries: 300 MPa at 4 °C: TV below the detection limit. 400 MPa at 4 °C: MNV-1 below the detection limit.	[104]
High-Pressure Homogenization	MNV-1, feline calicivirus-F9 and bacteriophage MS2	Blueberry juice	Intensity: 100–300 MPa. Treatment time: <2 s	4–5 log PFU/mL of FCV-F9 and MS2 (100 MPa). 0.71 log PFU/mL for MNV-1 (300 MPa).	[141]
HHP	HuNoV GI.1 and GII.4	Strawberries, blueberries, raspberries, and their purees	Intensity: 250 to 650 MPa (0 °C). Treatment time: 2 min	Highest reduction observed in blueberries: >3.2 log genomic copies/g with 550 MPa for GI.1 strain >4.1 log genomic copies/g with 300 MPa for GII.4 strain Purees: >2.9 log genomic copies/g with ≥550 MPa for HuNoV GI.1. >4.0 log genomic copies/g with ≥550 MPa for HuNoV GII.4.	[102]
HHP	O157 and Non-O157 Serogroups of Shiga Toxin-Producing *E. coli*	Blueberry juice	Intensity: 450 Mpa at 4 and 45 °C with or without antimicrobials and habituated strains for 3-days. After habituation at 4 °C: 0.5% of carvacrol and caprylic acid. After habituation at 45 °C: 0.1% of carvacrol and caprylic acid. Treatment time: 1–7 min	Effects of habituation and antimicrobials at 45 °C were less pronounced than at 4 °C. 450 MPa at 4 °C for 7 min Before habituation: 1.4 and 1.6 log CFU/mL for O157 and non-O157 serogroups. After habituation: 2.6 and 3.3 log CFU/mL for O157 and non-O157 serogroups. With carvacrol (after habituation): 4.2 log CFU/mL for *E. coli* O157. Without carvacrol (after habituation): 2.6 log CFU/mL for *E. coli* O157.	[105]
Pulsed electric field (PEF)	*E. coli* K12 and *Listeria innocua* inoculated and natural microbiota	Fresh blueberries	PEF: 2 kV/cm, 1 µs pulse width and 100 pulses per second, alone or in combination with 0.25–0.5% PAA. Treatment time: 2–4 min.	Increased efficiency of PEF + PAA. *E. coli* and *L. innocua*: up to 3 log CFU/g Native microbiota: 2 log CFU/g	[110]
PEF	*S. cerevisiae*, *E. coli*, and *S. aureus*	Blueberry juice	Voltage: 100–500 V. Pulse duration: 50–250 μs. Pulse number: 20–100 pulses.	Increased efficiency with voltage, pulse duration and pulse number. 500 V for 200 μs and 80 pulses: *S. cerevisiae*: 5.42 log CFU/mL *E. coli*: 5.32 log CFU/mL *S. aureus*: 4.77 log CFU/mL.	[109]
Cold Plasma (CP)	TPC and YMC	Fresh blueberries	Atmospheric CP: 15–120 s	TPC (4 ºC for 7 d): 1.5–2.0 log CFU/g compared to the control. CP did not significantly reduce YMC.	[115]
CP	TV and MNV-1	Fresh blueberries	Cold plasma alone with 4 cubic feet/minute (cfm) or with 7 cfm of ambient-temperature air. Treatment time: 15–120 s	Virus inactivation was dependent on treatment time. Addition of 7 cfm of ambient air: TV: 3.5 log PFU/g (120 s) MNV: >5 log PFU/g (90 s)	[116]
CP	TPC	Fresh blueberries	Microwave power: 9, 7, and 5 W; argon flow of 7 L/min. Treatment time: 15–60 s	At 5 and 7 W (30 s): >1 log.	[142]
CP	*Bacillus* sp.	Blueberry juice	CP at 11 kV and 1000 Hz Oxygen: 0, 0.5% and 1% Treatment time: 2–6 min	7.2 log (6 min *y* O_2_ 1%)	[119]
Ionizing radiation	YMC, strains of *Salmonella enterica* and *Listeria monocytogenes*.	Fresh blueberries (*Vaccinium corymbosum* cv. Bluecrop)	Gamma irradiation: 0.4 kGy	Irradiation did not significantly affect YMC. *Salmonella* and *L. monocytogenes* reduced in ~1 log CFU/g.	[123]
Ionizing radiation	*Toxoplasma gondii* oocysts	Fresh blueberries	Gamma radiation: 0.2–0.6 kGy at 4 °C.	All treatments produced reductions of 4 log PFU/g beyond the detection limit of 1 log PFU/g.	[124]
Ionizing radiation	*E. coli* K-12	Fresh blueberries (*Vaccinium corymbosum* cvs. Collins, Bluecrop)	Electron-beam: 0.5–3 kGy	*E. coli* K-12 gradually decreased with the increased dose. With 3.13 kGy: *E. coli*: ~8 log CFU/g at 3.13 kGy 72% decay at 4 °C, and 70% decay at room temperature.	[125]
Cavitation	TPC, YMC, and Heat-resistant mould	Blueberry Puree	Heating: at 40 °C (stage 1), at 80 °C (stage 2), after 10 min holding at 80 °C (stage 3), and pasteurization from 86 to 96 °C (stage 4) (continuous or steady state with 1 to 2 min holding time).	Heat-resistant moulds were inactivated at 94 to 96 °C within 1 to 2 min holding time. TPC in both slow and rapid collected samples at temperatures >86 °C were ≤10 CFU/g. YMC were undetectable for all samples. Blueberry purée products were shelf-stable for 24 week-storage at room temperature.	[143]
Ultrasound (US)	*Aspergillus ochraceus*, *Penicillium expansum*, *Rhodotorula* sp., *Saccharomyces cerevisiae,* and *Alicyclobacillus acidoterrestris.*	Apple, cranberry, and blueberry juice and nectar	Intensity: 20 kHz. Amplitude: 60–120 µm. Temperature: 20–60 °C. Treatment time: 3–9 min.	US treatments at 60 °C for 3, 6 and 9 min: 3.56–5.93 log CFU/mL for all yeasts and moulds The treatments were not very effective against *A. acidoterrestris*.	[129]
US	TPC, YMC and coliforms	Blueberry juice	Frequency: 20 kHz Flow: 24 mL/min or 93.5 mL/min. Amplitude: 40, 80, 100%.	US 93.5 mL/min/100 A: TAB: 1.36 log CFU/mL YMC: 1.18 log CFU/mL Coliforms: 1.28 log CFU/mL	[144]
Combination of US, heat, and pressure.	*Escherichia coli* O157:H7	Blueberry juice	Heat: 30–80 °C (10 min). 80 °C (5–20 min). Sonication: 280–700 W, 20 °C for 10 min 280–700 W, 30–60 °C for 10 min. TS: 40 °C, 560 W. MT: 350 MPa, 40 °C. MS: 560 W, 5 min/350 MPa. MTS: 560 W, 5 min, 40 °C/350 MPa, 40 °C for 5–20 min.	Heat inactivation at 80 °C > than the other temperatures 80 °C (10 min): 3.21 log CFU/mL. 80 °C (20 min): 4.32 log CFU/mL. The inactivation increased with increasing sonication power: 5.10 log CFU/mL (60 °C, 700 W). MTS and MS (5 min): 5.85 and 5.2 log CFU/mL, respectively.	[130]
US combined with antimicrobial compounds	*E*. *coli* K12 and *L*. *innocua*	Fresh blueberries	US: 1 MHz or 20 kHz alone or combined with citral 10 mM. Treatment time: 30 or 15 min	Citral + US 1 MHz (30 min): *E*. *coli* K12: 5.23 log CFU/g. Citral + US 20 kHz (15 min): *L*. *innocua*: ~3 log CFU/g.	[130]
Cooling	Serovars of *Salmonella enterica*	Southern highbush blueberries (mixture of “Farthing”, “Sweetcrisp”, and “Emerald”)	Forced-air cooling: 60–90 min. Hydrocooling with no sanitizer: 6 min. Hydrocooling with 150 ppm free chlorine: 6 min	Hydrocooling with sanitizer was the most effective treatment: >4 log CFU/g at day 0. Hydrocooling with sanitizer + 21 d storage: >5 log CFU/g.	[145]
